# Chemotype, maternal genotype, or field neighbors: what influences performance and resource allocation in a perennial plant species the most?

**DOI:** 10.1007/s00442-025-05767-4

**Published:** 2025-07-14

**Authors:** Dominik Ziaja, Rohit Sasidharan, Ruth Jakobs, Elisabeth J. Eilers, Caroline Müller

**Affiliations:** 1 Department of Chemical Ecology, Bielefeld University, Universitätsstraße 25, 33615 Bielefeld, Germany; 2 CTL-GmbH, Chemisch-Technologisches Laboratorium, Krackser Straße 12, 33659 Bielefeld, Germany

**Keywords:** Asteraceae, Gas chromatography–mass spectrometry, Specialized metabolites, Common tansy, Trait-based ecology

## Abstract

Plants have to allocate resources into their growth and reproduction but also in phytochemicals used in interactions with their environment. Some species display an extraordinarily high diversity of such phytochemicals, called chemodiversity. In stands with different neighbors, plot chemodiversity may be even higher and provide associational resistance. Little is known about trade-offs in investment into growth and reproduction versus chemodiversity in plant individuals growing in chemically different neighborhoods.

We investigated such trade-offs using the perennial *Tanacetum vulgare*, which shows differences in leaf terpenoid composition, forming distinct chemotypes. We planted plots consisting of five plants of one of five chemotypes (homogenous neighborhoods) and plots consisting of five different chemotypes (heterogenous) in a field. Plants within each block were offspring from different mother plants (here called maternal genotypes). Over 2 years, plant performance traits related to growth and reproduction were recorded and the leaf terpenoid profiles were measured once. Depending on the chemotype, plants had significantly different chances of flowering. Plants in heterogenous plots produced a higher maximum number of flower heads than those in homogenous plots. The maternal genotype explained some of the variance in growth- and reproduction-related traits. No significant correlations were found between the terpenoid functional Hill diversity (FHD) or its components (terpenoid richness, evenness, and structural disparity) and growth- and reproduction-related plant traits. Our results suggest that both the terpenoid chemotype of an individual and the chemodiversity of its neighborhood can impact reproduction-related traits, possibly driven by interactions of the individual with the environment and different resource allocation.

**Supplementary Information:**

The online version contains supplementary material available at 10.1007/s00442-025-05767-4.

## Introduction

High biodiversity is considered to enhance functional stability of ecosystems and resistance towards environmental challenges (Isbell et al. 2017; Aubree et al. 2020). In particular, plant species-rich communities have been suggested to suffer less from herbivore damage than species-poor communities or monocultures due to associational resistance (Plath et al. 2012; Tahvanainen and Root 1972). Moreover, plant-species-rich communities can affect mutualistic interactions positively, indicated, for example, by a reciprocal relationship between flower abundance and number of flower visits by pollinators (Sutter et al. 2017). However, evidence on such beneficial impacts is mixed (Mutz et al. 2022; Tooker and Frank 2012). Also within species, phenotypic traits often differ due to genetic differentiation, which may have similar effects on ecosystem functions as species biodiversity. For example, intraspecific plant genotypic diversity impacts herbivore and predator abundances (Koricheva and Hayes 2018; Tooker and Frank 2012), and increased genotypic diversity can enhance aboveground production within plant species similar to the way as diversity across species (i.e., biodiversity), at least temporally (Cook-Patton et al. 2011; Prieto et al. 2015). Furthermore, the maternal genotype can influence growth and reproduction-related traits of the offspring due to different capacities in interacting with environmental conditions, such as, for example, the exploitation of nutrients (Roach and Wulff 1987). Chemical diversity, also called chemodiversity, can be considered as an important phenotypic trait of plants, which has pivotal functions in shaping interactions between plants and their environment (Müller and Junker 2022; Walker et al. 2022; Wetzel and Whitehead 2020) and depends on the genotype (Dussarrat et al. 2023). However, little is known about the relationships between intraspecific chemodiversity and biomass production within species.

Several hypotheses have been postulated why plants may produce so many different metabolites simultaneously and why a high intraspecific chemodiversity is evolutionarily maintained (Whitehead et al. 2021; Thon et al. 2024). For example, the synergy hypothesis proposes that compounds may act synergistically, increasing the effectiveness of individual plant defense metabolites (Richards et al. 2016). More chemodiverse plants may thus be visited by a higher diversity of herbivores, but nevertheless experience less herbivory, resulting in a higher fitness, as found in different *Piper* species (Richards et al. 2015). Moreover, chemodiversity of individual plants may change in response to attack by antagonists, which leads to the induction of several compounds such as terpenoids (Kaplan et al. 2008). The interaction diversity hypothesis states that chemodiversity results from the number of antagonistic and mutualistic interactions of a plant (Kessler and Kalske 2018). Apart from the chemodiversity of the individual itself, the local spatial distribution of chemodiversity may be an important determinant for biotic interactions and plant fitness. For example, plants of *Brassica oleracea* growing in phytochemically more diverse conspecific neighborhoods were overall larger and less damaged, although harboring more herbivores, compared to plants growing in less diverse patches (Bustos-Segura et al. 2017). Density, frequency, or diversity of neighbors can modulate trophic interactions of focal plants via such associational effects, influencing plant defense traits (Mutz et al. 2022). These impacts strongly depend on the identity of the neighbor. For example, focal plants of *Oenothera biennis* had overall a lower fitness when grown together with conspecifics versus heterospecifics. Selection on plant biomass was particularly strong in patches with conspecifics of the same genotype compared to genotype mixtures of *O. biennis* (Parachnowitsch et al. 2014). Whether the performance of a plant individual is affected by the chemodiversity of its neighbors has received little attention.

Investments into specialized defense metabolites, which influence the chemodiversity, can impose different types of costs. Ecological costs arise when specialized metabolites have adverse effects on potential mutualists (Koricheva 2002). Allocation costs may arise under limited resources, causing trade-offs between investment into growth, reproduction, or defense (Züst and Agrawal 2017). Whether plants prioritize growth or defense can depend on the environmental conditions and on the individual strategies a plant uses to cope with these conditions (Koffel et al. 2018; MacTavish and Anderson 2020; Hahn et al. 2021). Elements needed for the biosynthesis of specialized metabolites depend on the skeleton of the given metabolite as well as on the processes involved in its synthesis. For example, terpenoids are carbon (C)-based compounds, but also require nitrogen (N) for enzymatic pathways and storage (Hamilton et al. 2001). The biosynthesis of terpenoids is also considered to be costly, because it does not share enzymes with other metabolic pathways (Gershenzon 1994). The diversification of terpenoids can be enhanced by promiscuous enzymes that are able to synthesize multiple products from a single substrate (Degenhardt et al. 2009). How metabolic costs for a diversity of terpenoids in highly chemodiverse plant species potentially trade off with growth and reproduction of plant individuals has so far been understudied.

*Tanacetum vulgare* (Asteraceae; common tansy) is a perennial species with an exceptional high diversity in terpenoid composition (Clancy et al. 2016; Keskitalo et al. 2001; Wolf et al. 2012). Different chemotypes can be discriminated based on the dominant leaf terpenoids (Holopainen et al. 1986). In the field, certain chemotypes seem to be more abundant than others, but even within local areas, more than a dozen different chemotypes can co-occur (Kleine and Müller 2011). Therefore, visiting organisms experience different chemodiversity between neighboring plants but also on the patch level depending on the composition of chemotypes. Different herbivore species show distinct preferences for certain chemotypes (Clancy et al. 2016; Jakobs and Müller 2019; Kleine and Müller 2011) and may thus also drive changes in chemotype frequencies within patches of *T. vulgare* individuals. Moreover, plants of distinct chemotypes differ in the duration of their blooming period and pollen nutritional quality, thereby affecting florivores (Eilers et al. 2021; Sasidharan et al. 2023), and thus potentially also pollinators and reproductive success. However, to our knowledge, insights are lacking whether individuals of distinct chemotypes or plots of *T. vulgare* differing in chemodiversity show ontrasting growth and reproduction.

Here, we set up a common garden field experiment to assess the effects of intraspecific chemodiversity on plant individual and plot performance, i.e., growth- and reproduction-related traits. The common garden contained 60 plots with each plot consisting of five offspring plants derived from different maternal genotypes (likely different genotypes) either of the same chemotype (homogenous plots) or different chemotypes (heterogenous plots). We hypothesized that individuals of different chemotypes differ in growth- and reproduction-related traits. Plants grown in heterogenous plots were expected to produce more biomass, flower heads and seeds than individuals growing in homogenous plots, potentially due to lower overall herbivory. Similarly, on the individual level, plants with a higher leaf terpenoid chemodiversity were expected to produce more aboveground shoot and seed biomass, but due to trade-offs invest less in other growth- and reproduction-related traits. Moreover, when growing in heterogenous plots, plants of individual chemotypes were expected to be chemically less diverse than when growing in homogenous plots, potentially due to lower infestation rates and thus lower induction of terpenoids. A relationship between terpenoid chemodiversity and leaf C and N content was expected but with unclear direction, since multiple hypotheses on allocation costs of terpenoids exist.

## Materials and methods

### Rearing of experimental plants and common garden setup

Plants were grown from seeds that had been collected from 14  *T. vulgare* maternal plants (here called “maternal genotypes”) growing at a distance of at least 20 m spread across four sites close to Bielefeld, Germany, assuming that these are different maternal genotypes. At 6–8 weeks of age, the apical 3 cm of the second-youngest leaf was harvested from each plant and the terpenoid profile determined (see below). Since *T. vulgare* is outcrossing, the offspring of maternal genotypes usually belong to different chemotypes. Plants belonging to in total five distinct terpenoid profiles (chemotypes) were chosen. These chemotypes were determined based on the most dominant terpenoid(s) that contributed to >50% of the total terpenoid composition. Two chemotypes were mono-chemotypes, in which just one terpenoid contributed to >50%, namely artemisia ketone (called Keto chemotype from here on) and β-thujone (Bthu chemotype). In the other three chemotypes, two to three terpenoids made up >50%, namely α- and β-thujone (ABThu chemotype), artemisyl acetate, artemisia ketone and artemisia alcohol (Aacet chemotype), or (*Z*)-myroxide, santolina triene and artemisyl acetate (Myrox chemotype). From each of 12 of the 14 maternal genotypes, offspring of two distinct chemotypes could be collected, while from two maternal genotypes, offspring of three distinct chemotypes could be gained. From each maternal genotype–chemotype combination, three to eight plants were taken. In total, we established a stock of 150 plants from this offspring population.

Plants were grown in a 1:1 mixture of sterilized potting soil and river sand. When plants had sufficient biomass, two clones per stock plant (total of 300 plants) were produced from rhizomes, grown in the greenhouse at 21 °C and 16 h: 8 h, light: dark, and fertilized weekly (as in Kleine and Müller 2014). From September 2019 onwards, the pots were placed outside in a sand bed for acclimatization and kept there until the last week of May 2020 when they were transferred into a common garden field site.

The site was 17 × 24 m large and located in Bielefeld (52°03′39.43′N, 8°49′46.66′E; elevation 142 m; for details, see Ziaja and Müller 2023). The *T. vulgare* plants were planted in six blocks with a spacing of 2 m between blocks in the ploughed ground (100% clay with horse manure incorporated in 2018). Each block contained ten plots of 1 m × 1 m with 1 m spacing between plots. Each plot contained five plants planted within the plot with a distance of 40—65 cm between plants. In half of the plots, all five plants belonged to the same chemotype (= homogenous plots, n = 6 plots per chemotype), and in the other half, the five plants belonged to different chemotypes (= heterogenous, n = 30 plots). The two clones per plant individual were always planted in the same block, with one clone per plot-type, allowing us to test phenotypic plasticity in dependence of the neighborhood. Within each plot, all plants originated from different maternal genotypes and each plant was grown in the soil within a tube of 25 cm length and 16 cm diameter to separate plants from each other and prevent root communication and competition between plants. The nutrient composition and water content in the soil across the experimental field site were relatively homogenous (Supplement Table S1).

## Determination of growth- and reproduction-related plant traits

Growth and reproduction-related plant traits were recorded in 2020 and 2021. As growth-related traits, plant height, the number of shoots, the number of leaves, and the dry aboveground biomass were recorded and measured. Plant height (from ground to top of the highest shoot), number of shoots, and number of leaves were recorded in the fourth week in August 2020, the first week in April and third week in June 2021. To determine the dry aboveground biomass, all plants were cut down to a height of 5 cm above ground between end of November and mid-December 2020, dried for 48 h at 60 °C and weighed. The bloom onset, the maximum number of flower heads, and the total seed dry biomass were taken as reproduction-related traits. For bloom onset, the day at which the first plant had at least one fully opened flower head was set as day zero in each of the two years 2020 and 2021. The delay in flowering (in number of days) per individual plant was then monitored as bloom onset trait. The number of flower heads was counted from end July (2020) or beginning August (2021) until the third (2020) or fourth week (2021) of September and the maximum number of flower heads per plant in each year taken for further analysis. To determine the total seed biomass per plant, flower heads with ripe seeds were subsequently collected thrice a week from mid-September until mid-December 2020, dried, and stored in desiccators. Seeds collected from each plant were then pooled and weighed.

For later analyses of terpenoids, metabolic fingerprints, and C and N content, the apical 4 cm of the second-youngest leaf of each of two stems per plant was sampled once in mid-June 2021, frozen in liquid nitrogen, and stored at −80 °C. Samples were then freeze-dried and homogenized. A subsample of about 4 mg dw was taken for analysis of the C and N content after combustion in a C–N analyzer (Vario MICRO Cube, Elementar Analysensysteme, Hanau, Germany). For an overview of all taken plant traits, see Table 1.

**Table 1:** Overview of growth and reproduction-related plant traits measured from *Tanacetum vulgare* plants grown in a common garden field in the years 2020 and 2021

Phenotypic trait	Category	Season 2020	Season 2021	Aug 2020	April 2021	June 2021
Dry plant biomass (g dw^a^)	Growth	x				
Number of leaves	Growth			x	x	x
Number of shoots	Growth			x	x	x
Plant height (cm)	Growth			x	x	x
Total seed biomass (g dw)	Reproduction	x				
Maximum count flower heads	Reproduction	x	x			
Bloom onset (in d, after first flowering plant)	Reproduction	x	x			
N content (%)	Biochemical					x
C content (%)	Biochemical					x

^a^dw: dry weight

## Determination of leaf terpenoid profiles

For determination of terpenoid profiles, about 10 mg of dried leaf material were extracted in 1 mL *n*-heptane (Roth, 99% HPLC grade) by sonication for 5 min. After centrifugation, the supernatants were analyzed using gas chromatography coupled with mass spectrometry (GC–MS, GC 2010plus—MS QP2020, Shimadzu, Japan; column VF-5 MS, 30 m length, 0.2 mm ID, with 10 m guard column, Varian, United States) in electron impact ionization mode at 70 eV. Helium was used as carrier gas with a column flow of 1.5 mL min^−1^. The GC injection port was kept at 240 °C and operated in a 10:1 split mode. The initial GC temperature was set to 50 °C, kept for 5 min, increased to 250 °C at a rate of 10 °C min^−1^ and further increased to 280 °C at a rate of 30 °C min^−1^, hold for 3 min. After each batch of 23 samples, a blank containing the solvent and internal standard was measured to identify and subtract potential contaminations. In addition, an alkane standard mix (C7–C40, Sigma Aldrich, Germany) was analyzed with the same GC–MS method to calculate retention indices (RI) (van den Dool and Kratz 1963). Compounds were identified by comparing the RI and mass spectra with those of synthetic reference compounds, where available, as well as with library entries of the National Institute of Standards and Technology NIST 2014, Pherobase (El-Sayed 2012) and mass spectra and RI values reported in Adams (2007). Compound quantification was based on the total ion chromatogram of peaks and the relative composition of the terpenoids was calculated.

## Determination of leaf metabolic fingerprints

A subset of 181 samples (at least 35 samples per chemotype, 9 per maternal genotype) was analyzed using an ultra-high performance liquid chromatograph coupled to a quadrupole time-of-flight mass spectrometer (UHPLC-QTOF-MS/MS; UHPLC: Dionex UltiMate 3000, Thermo Fisher Scientific, San José, CA, USA; QTOF: compact, Bruker Daltonics, Bremen, Germany), equipped with a Kinetex XB-C18 column (150 × 2.1 mm, 1.7 µm, with guard column; Phenomenex), as described in Dussarrat et al. (2023). Compounds were separated at a flow rate of 0.5 mL min^−1^ using Millipore water with 0.1% formic acid (eluent A) and acetonitrile with 0.1% formic acid (eluent B), at a gradient from 2 to 30% eluent B in the first 20 min, continuing from 30 to 75% eluent B within 9 min, ending with column cleaning and equilibration. Measurements took place in negative electrospray ionization mode with an *m*/*z* range of 50–1300 and a spectra rate of 6 Hz. Further instruments settings were used as in Schweiger et al. (2021). Auto MS/MS was used with the isolation width and collision energy being ramped along with increasing *m*/*z*. For selected samples, MS/MS analyses were performed to target selected ions with multiple reaction monitoring. Chromatograms were recalibrated along the *m*/*z* axis using a sodium formate calibration solution and processed with DataAnalysis (v. 4.4, Bruker Daltonics). Features with a retention time between 1.25 and 29 min were picked, grouped together by bucketing and quantified as described in Dussarrat et al. (2023). Only features present in at least two samples and with mean peak intensities higher than 50 times compared to the blanks were retained in the data set and metabolite richness determined per sample.

## Statistics

All statistical analyses were conducted with R, version 4.2.1 (R Developmental Core Team 2022), using the packages dplyr (Wickham et al. 2021), glmmTMB (Brooks et al. 2017), coxme (Therneau 2024), ggplot2 (Wickham 2016), rstatix (Kassambra 2023), vegan (Oksanen et al. 2022), DHARMa (Hartig 2021), car (Fox and Weisberg 2019), insight (Lüdecke et al. 2019), Hmisc (Harrell 2023), emmeans (Lenth 2021), and pgirmess (Giraudoux 2022).

To analyze the effect of plot-type on the individual plant level, we applied pairwise tests to all recorded plant traits, allowing us to specifically compare the two clones per offspring plant growing under the two distinct conditions (homogenous or heterogenous). If a plant trait was normally distributed (Shapiro–Wilk test) a pairwise t test was applied, if not, a pairwise Wilcoxon test was applied. In addition, generalized linear mixed models (GLMMs) were performed to test effects on recorded plant traits except bloom onset on the individual plant level. For these GLMMs, chemotype, plot-type, and the chemotype x plot-type interaction were implemented as fixed effects; plot nested in block, the clone ID, and maternal genotype were set as random effects. Due to zero-inflation of the dry total seed biomass, this trait was analyzed using a gamma-hurdle model. For all GLMMs, the goodness of fit and appropriate distribution (Gaussian, gamma, Poisson, and negative binomial 1) were evaluated based on simulated residuals using DHARMa plots. Due to its character as time-to-event data, bloom onset was analyzed using mixed-effects cox models with identical fixed and random-effects specifications as in the GLMMs. Whether fixed effects had a significant effect was tested on all models using type 2 Wald chi-square tests.

To assess chemodiversity also in form of a metric gradient, the terpenoid diversity was quantified using the functional Hill diversity. The diversity index is defined by

$${}^{q}\text{FHD}\left(Q\right)= {\left[\sum_{i=1}^{S}\sum_{j=1}^{S}{d}_{ij}{\left(\frac{{p}_{i}{p}_{j}}{Q}\right)}^{q}\right]}^{1/\left(1-q\right)},$$

where p_*i*_ and p_*j*_ are the relative abundances of compounds *i* and *j*, d_*ij*_ is the dissimilarity between the compounds *i* and *j*, *Q* is Rao’s Q, and *q* controls the sensitivity of the measure to the relative abundances of the compounds (Petrén et al. 2023). Our analysis was conducted with q = 1, meaning that compounds were weighed into the calculation in proportions of their abundances. On the individual plant level (FHD_ind_), the index was calculated based on the relative abundance of the compounds per plant, whereas on the plot level (FHD_plot_), it was based on the average relative abundance of the compounds per plot. This FHD_plot_ is ecologically relevant for an insect moving within a plot and receiving the terpenoid profiles of various plants in the nearest neighborhood. For further analysis, FHD_ind_ was deconstructed into its individual components, namely, the compound richness, evenness, and disparity, according to the developed guidelines (Petrén et al. 2023).

The relationships between the FHD components and the plant traits were examined using correlations. A Mantel test between the relative abundance of terpenoids and all recorded plant traits based on Bray–Curtis dissimilarity distance matrices was calculated. For a more pinpointed approach, pairwise Pearson correlations between FHD_ind_ as well as its components and the plant traits were calculated; to account for the number of correlations performed, a False Discovery Rate correction was applied. For both correlation analysis, the data was ln+1 transformed. To examine how chemodiversity behaves across different matrices, a Pearson correlation and a Mantel test based on Jaccard-distance matrices were calculated between the terpenoid richness (GC–MS data) and the metabolite richness (fingerprints, LC–MS data).

## Results

### Effects of chemotype and plot-type on growth- and reproduction-related plant traits

**Fig. 1:**
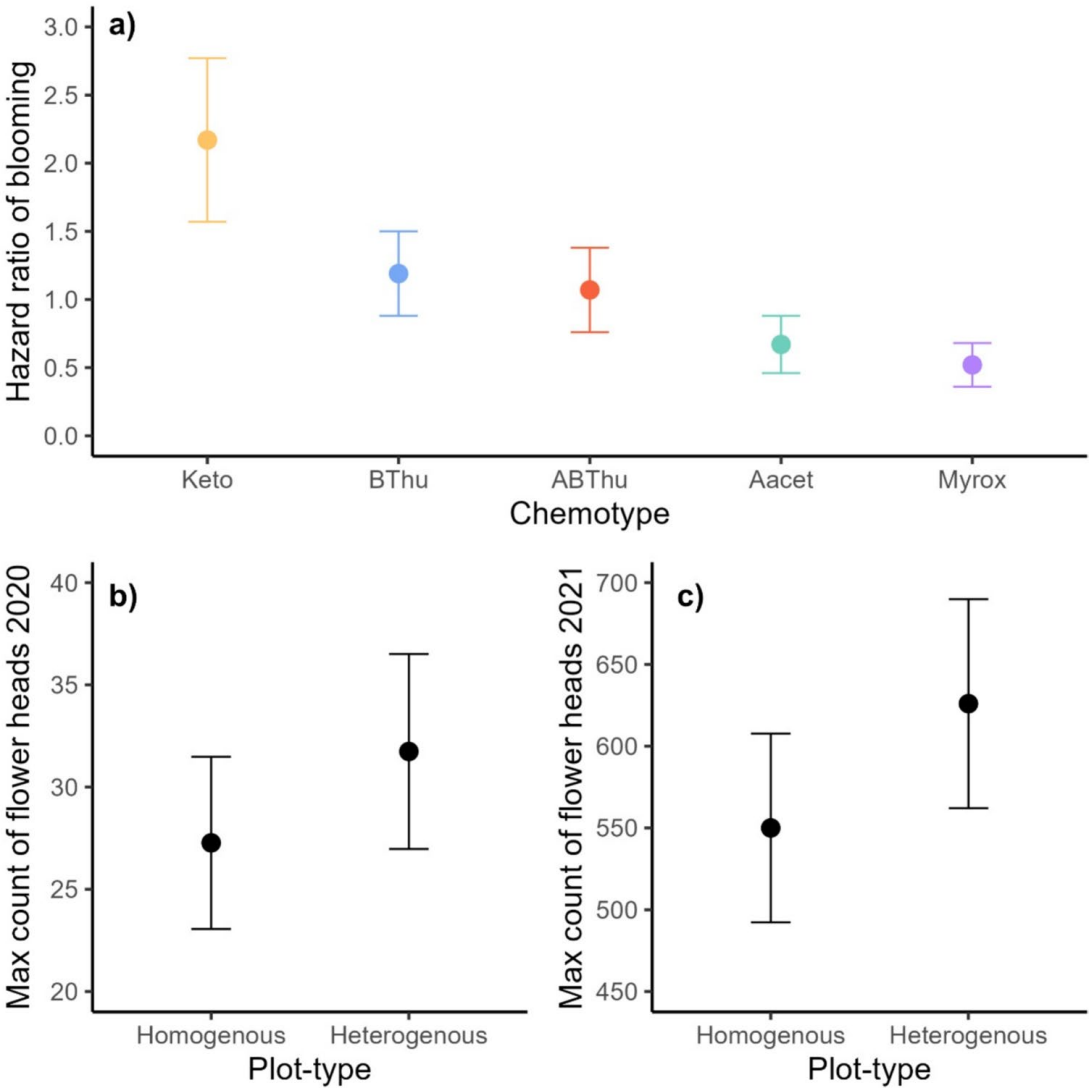
Estimated marginal means ± standard error of the hazard ratio of blooming (**a**) and the maximum count of flower heads in 2020 (**b**) and 2021 (**c**) of *Tanacetum vulgare* grown in plots of five individuals of the same (homogenous) or different (heterogenous) chemotypes. The different chemotypes Keto, BThu, ABThu, and Myrox refer to the foliar terpenoid profile of the plants. Means were estimated for the significant plant trait–fixed effects combinations (Table 2) in a mixed-effects cox model (**a**) or in generalized linear mixed effect models (**b**, **c**). Please note the different Y-axis for the maximum count of flower heads in 2020 (**b**) and 2021 (**c**)

All growth-related plant traits, i.e., dry plant biomass, number of leaves, number of shoots and plant height as well as C and N contents did not differ significantly between chemotypes, plot-types, or their interactions (Table 2). Reproduction-related traits of the plant individuals differed significantly between chemotypes or plot-types (Table 2). Bloom onset in the first year was the only trait that differed among chemotypes (Table 2, Fig. 1a). Plants of the Keto chemotype had a twofold higher chance of flowering compared to plants of the ABThu and BThu chemotype and an at least threefold higher chance compared to the Aacet and Myrox chemotype (Fig. 1a). Half of the Keto and BThu chemotype plants flowered after 18 ± 8.9 (median ± absolute deviation) days, whereas the median time until flowering for the ABThu, Aacet, and Myrox chemotype was 21 ± 7.41 days. Plants grown in homogenous plots displayed on average a lower maximum number of flower heads in 2020 and 2021 than those grown in heterogenous plots (Fig. 1b,c). The maternal genotype was an important factor as it explained relatively high proportions of the total variance for the plant height (11.7%), the number of leaves in June 2021 (7.5%), as well as the maximum number of flower heads in 2020 (10.8%) and 2021 (7.8%) (Table 2). Paired tests between the two clones growing in different neighborhoods (homogenous vs. heterogenous plots) revealed no significant differences (Supplement Table S2).

**Table 2:** Model estimates of phenotypical traits of *Tanacetum vulgare* plants grown in a common garden field in Bielefeld in the years 2020 and 2021

Response variables	Fixed effects			Random effects			
	Chemotype	Plot-type	Chemotype x plot-type	Block	Plot	Clone ID	Maternal genotype
	d.f. = 4	d.f. = 1	d.f. = 4	d.f. = 1	d.f. = 1	d.f. = 1	d.f. = 1
Dry plant biomass (gam^a^)	1.22	1.35	7.18	*5.59*	*4.43*	*15.18*	<*0.01*
Number of leaves, Aug 2020 (po^b^)	1.08	2.23	3.13	*2.54*	*18.10*	*33.04*	*5.81*
Number of leaves, Apr 2021 (po)	2.43	0.18	3.10	*26.92*	*16.16*	*27.83*	*4.37*
Number of leaves, Jun 2021 (po)	6.90	0.56	3.51	*7.79*	*23.41*	*46.62*	*7.49*
Number of shoots, Aug 2020 (po)	7.63	0.22	2.60	<*0.01*	*1.04*	*8.19*	*3.50*
Number of shoots, Apr 2020 (po)	3.01	0.86	0.82	*22.72*	*7.01*	*4.84*	*4.31*
Number of shoots, Jun 2021 (po)	2.14	1.43	2.14	*0.86*	<*0.01*	<*0.01*	*1.97*
Plant height, Aug 2020 (gaus^c^)	7.06	2.81	1.45	*4.25*	<*0.01*	*25.87*	*3.25*
Plant height, Apr 2021 (gaus)	6.62	0.89	7.04	*8.86*	*3.80*	*19.37*	*7.62*
Plant height, Jun 2021 (gaus)	1.76	0.19	5.34	*3.49*	*8.88*	*23.26*	*11.70*
Total seed dry biomass	5.42	3.66	2.18	*2.21*	*4.91*	<*0.01*	<*0.01*
Zero-truncated hurdle GLMM (gam)	5.71	0.40	3.44				
Max count flower heads 2020 (nb1^d^)	4.51	4.38*	4.42	*2.41*	<*0.01*	*13.68*	*10.78*
Max count flower heads 2021 (nb1)	3.03	3.91*	1.78	*1.82*	*0.00*	*11.03*	*7.81*
Bloom onset 2020 (coxme^e^)	**10.44**	0.16	3.25	—	—	—	—
Bloom onset 2021 (coxme)	6.23	0.41	1.99	—	—	—	—
N content (gaus)	8.15	0.02	2.05	*17.52*	*4.65*	*8.51*	*0.66*
C content (gaus)	3.68	0.07	4.10	*2.09*	*1.81*	<*0.01*	*2.67*
C-to-N ratio (gaus)	8.03	0.06	3.62	*17.45*	*5.39*	*7.84*	*0.30*

Bloom onset was modeled using mixed-effects cox models, all other response variables were modeled using GLMMs. Shown are the *Χ*^*2*^ estimates based on Wald’s type 2 Χ^2^ test (regular font) and the proportion of variance explained by random effects (italic). Numbers in bold indicate significant effects with asterisks displaying the significance level (*p < 0.05; **p < 0.01; ***p < 0.001). For the zero-truncated hurdle model, estimates of the conditional compartment are displayed in the first line, estimates of the zero compartment in the second line. Random effect variances for bloom onset are marked with “**—**”, because these were modeled with mixed-effects cox models, not enabling the extraction of all variance components. ^a^gam: response variable was modeled using a Gamma distribution, ^b^po: response variable was modeled using a Poisson distribution, ^c^gaus: response variable was modeled using a Gaussian distribution, ^d^nb1: response variable was modeled using a negative binomial 2 distribution, ^e^coxme: response variable was modeled using mixed-effects cox model, not GLMMs

## Functional Hill diversity (FHD) on individual plant and plot level

**Fig. 2:**
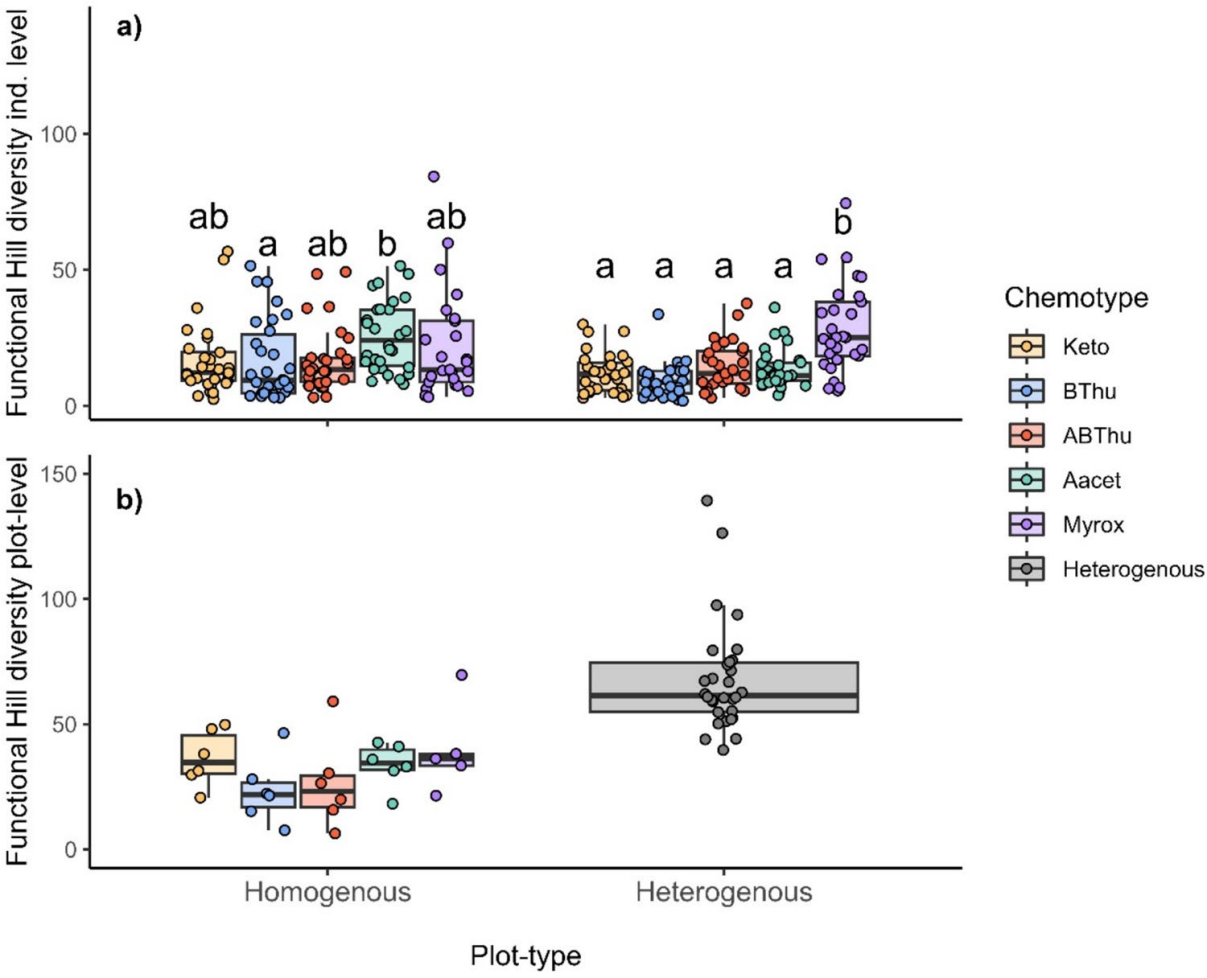
Functional Hill diversity calculated based on the relative terpenoid composition of each individual plant (**a**, *n* = 25–30) or averaged per plot (**b**, *n* = 5–30) of *Tanacetum vulgare* plants grown in plots of five individuals with the same (homogenous) or different chemotypes (heterogenous) in a common garden field. Chemotypes were dominated by either artemisia ketone (Keto), β-thujone (BThu), α- and β-thujone (ABThu), artemisyl acetate, artemisia ketone, and artemisia alcohol (Aacet) or (*Z*)-myroxide, santolina triene, and artemisyl acetate (Myrox). Different letters indicate significant differences based on Kruskal–Wallis multiple comparisons (*p* ≤ 0.05) calculated for the individual plant-level after an overall significant difference was revealed by a Kruskal–Wallis test (*Χ*^*2*^ = 55.90, *p* < 0.001)

The FHD_ind_ differed between plants of different chemotypes. In homogenous plots, plants of the Aacet chemotype had the highest FHD_ind_ and plants of the BThu chemotype the lowest. In contrast, in heterogenous plots, the FHD_ind_ was significantly higher in plants of the Myrox chemotype compared to the other chemotypes (Fig. 2a). Moreover, in plants of the Aacet chemotype, FHD_ind_ was significantly higher in plants growing in homo- compared to heterogenous plots. On the plot level, heterogenous plots displayed a higher FHD_plot_ than all homogenous plots (Fig. 2b). Within the homogenous plots, the Keto, Aacet, and Myrox chemotype had a comparably higher FHD_plot_ than the Bthu and ABThu chemotypes (Fig. 2b).

## Correlations between chemodiversity and growth- and reproduction-related plant traits

**Fig. 3:**
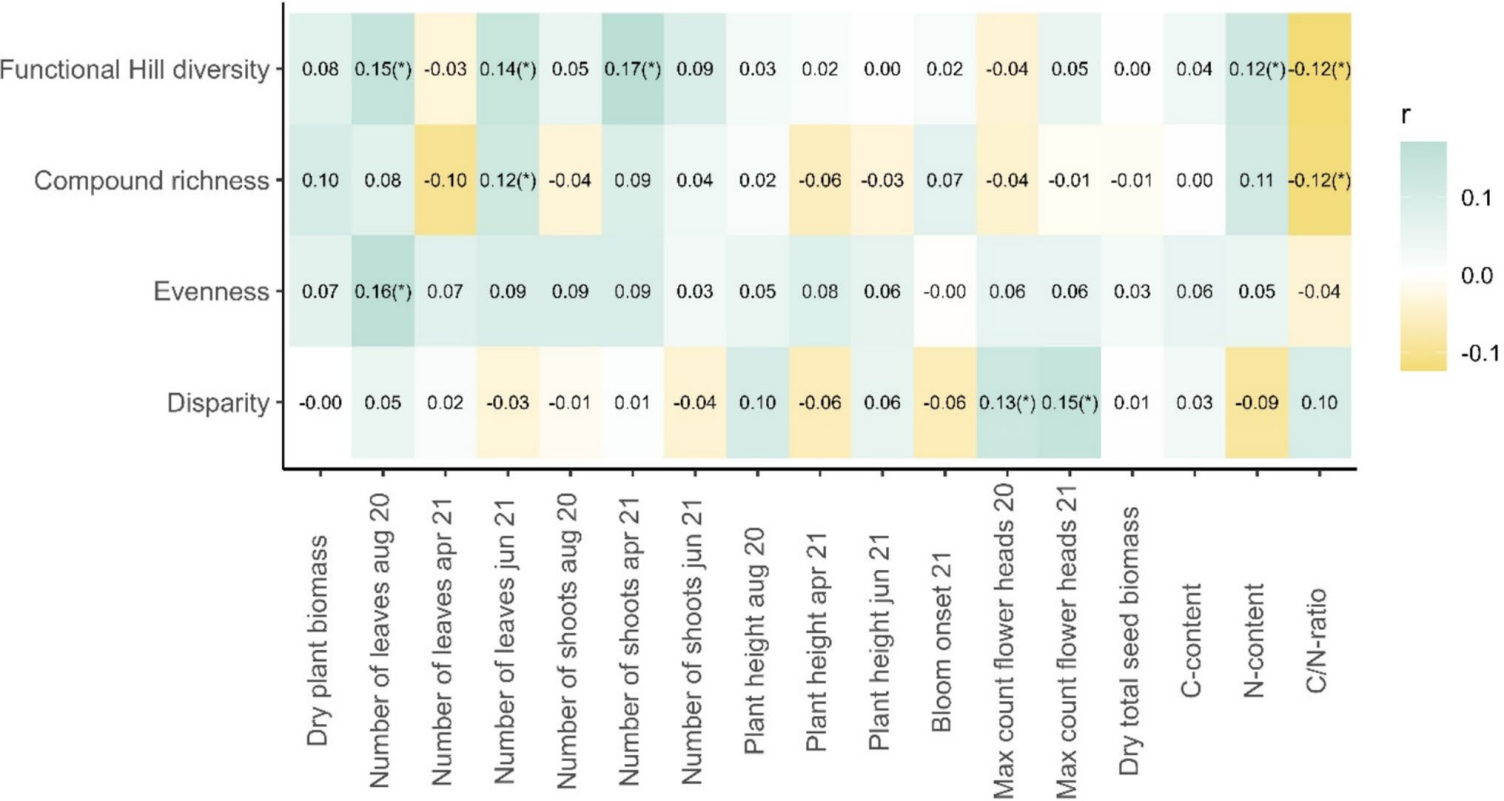
Pearson correlation of the functional Hill diversity and each of its components with every recorded growth and reproduction-related plant trait (ln+1-transformed) of *Tanacetum vulgare* plants grown in a common garden field in 2020 and 2021. Numbers in tiles display the regression coefficient. After adjustment of *p* values using false discovery rate, no significant correlations were found; correlations that were significant before *p* adjustment are marked with “(*)”. The functional Hill diversity was calculated based on the leaf terpenoid profile of each plant

In contrast to the analysis of the two distinct plot-type classes, no significant correlations were found between the different diversity indices and the measured growth- and reproduction-related plant traits (Fig. 3). However, correlations significant before *p *value adjustment still indicated trends. Here, a positive correlation of FHD_ind_ with the number of leaves in August 2020 and June 2021, the number of shoots in April 2021, and the N content as well as a negative correlation with the C-to-N ratio were suggested (Fig. 3). Three of these trends were also found among the FHD_ind_ components (compound richness, evenness, and disparity). The terpenoid richness indicated a positive relationship with the number of leaves in June 2021 and the evenness with the number of leaves in August 2020, while it was negatively correlated with the C-to-N ratio. The disparity suggested positive correlations with the maximum number of flower heads in both years, while FHD_ind_ did not (Fig. 3).

**Fig. 4:**
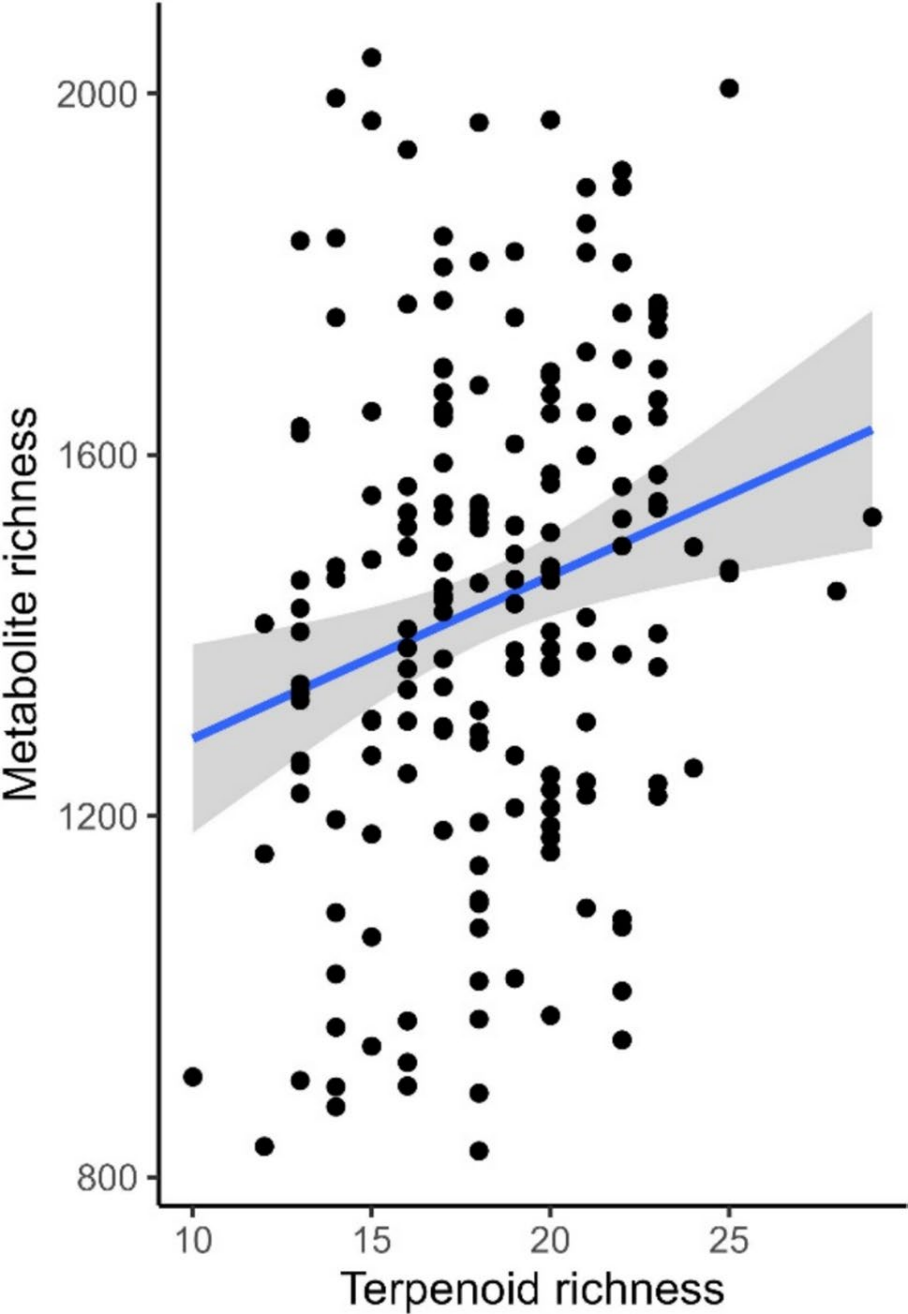
Relationship between terpenoid richness (measured by GC–MS) and metabolite richness (measured by UHPLC-QToF-MS/MS) in young leaves of Tanacetum vulgare plants grown in a common garden field, analyzed in June 2021. The blue line displays predicted values of the linear regression of the form: metabolite richness = (18.03 × terpenoid richness) + 1105.32; *r* = 0.044, *p* = 0.003. Gray areas represent the 95% confidence intervals

When comparing diversity indices between the different types of metabolites (analyzed by the two analytical platforms), a significant correlation between leaf terpenoid and metabolite richness was revealed (Pearson: *r* = 0.22, *p* = 0.003; Mantel test: *r* = 0.04, *p* = 0.001; Fig. 4).

## Discussion

Our results revealed a significant impact of the chemotype as well as the heterogeneity of chemotypes in the neighborhood on reproduction-related plant traits. However, the chemodiversity index (FHD) and its components did not show any significant correlations with different growth- and reproduction-related traits, but trends of positive correlations were found. Next to chemodiversity, the maternal genotype contributed to the variance of several plant traits, such as plant height, number of leaves in 2021, and maximum number of flower heads in both years.

We hypothesized that individual plants would differ in growth and reproduction depending on both chemotype and plot-type. Significant effects were not found for growth-related but for reproduction-related plant traits for both chemotype and plot-type. Depending on the chemotype, plants had a significantly different chance of flowering in 2020. Such chemotype-specific blooming behavior has also been reported in a laboratory and a field study with *T. vulgare* and may arise due to chemotype-specific competitive abilities (Eilers et al 2021; Ojeda-Prieto et al. 2024). The lack of a chemotype effect on growth and all other reproduction-related plant traits is surprising, because significant effects of plant chemotype were found on the abundance of aphids specialized on *T. vulgare*, the main herbivores on this plant species, from May to July in this same common garden experiment in 2020 and 2021 (Ziaja and Müller 2023). Likewise, in various other studies, the individual plant chemotype was shown to affect herbivory (Kleine and Müller 2011; Balint et al. 2016; Jakobs and Müller 2018), herbivore damage (Bustos-Segura et al. 2017), and florivore preferences (Eilers et al. 2021; Sasidharan et al. 2023), which should influence plant growth and reproduction. However, chemotypes may have differed in other growth- or reproduction-related traits, not measured in the present study, in response to aphid infestation. For example, in soybean [*Glycine max* (L.) Merril] not seed yield, but seed number and seed oil concentrations were significantly reduced when plants were infested with aphids and effects on plant size depended on the growth stage at which aphid infestation took place (Riedell and Catangui 2006).

Besides distinct chemotype-specific biotic impacts on different plant individuals, different metabolic costs of the chemotypes were also expected to affect growth and fitness in *T. vulgare*. The fact that different chemotypes did solely differ in flowering behavior but not in growth and reproduction traits may be due to similar metabolic costs for the synthesis of different terpenoids in *T. vulgare*. Monoterpene and sesquiterpene synthases can be promiscuous, forming multiple products from single substrates (Degenhardt et al. 2009). Especially, because some chemotypes involved in this study share the same or at least structurally similar main terpenoids, synthesis costs may not differ much between chemotypes. For example, both chemotypes ABThu and BThu have high proportions of the structurally similar α-thujone and/or β-thujone. Differential metabolic costs may become more apparent under nutrient- or water-restricted conditions. For example, drought is known to impose chemotype-specific changes in terpenoid profiles of *T. vulgare* (Kleine and Müller 2014). However, in the common garden field, in which the experiment was conducted, plants did not suffer from resource scarcity.

For plot type effects, we had expected a higher growth and reproduction for plants growing in heterogenous compared to homogenous plots due to a higher surrounding chemodiversity and thus lower herbivore damage, as previously observed in other plant species (Salazar et al. 2016; Bustos-Segura et al. 2017). Contrary to these expectations, our results showed that *T. vulgare* plants in homogenous and heterogenous neighborhoods produced a similar biomass and had a comparable reproductive output (i.e., total seed biomass), but plants in heterogenous plots had a higher maximum number of flower heads in both years. These plants growing in heterogenous plots may have to allocate more resources into potential reproduction by producing more flower heads due to ecological costs. The more diverse, mixed odor bouquet may have adverse effects on plant localization by insects, including mutualists such as pollinators (Webster and Cardé 2017), which could result in a lower seed set if not compensated by an increased number of flower heads. In *T. vulgare*, the leaf and flower terpenoids are similar to a large extent, allowing grouping in the same chemotypes (Sasidharan et al. 2023). Recently it has been shown that plot-type can also influence pollinator visitation, with more visitors in heterogenous than homogenous plots of *T. vulgare* plants, which also positively correlated with germination rates. In contrast, florivores were more affected by the chemotype, not plot-type (Sasidharan et al. 2024).

Next to the plot-type, the maternal genotype explained some of the variance for various growth- and reproduction-related traits. Effects of the genotype have likewise been shown in several plant species. For example, rice genotypes differed significantly in plant height and biomass (Shrestha et al. 2021) and various flower traits were found to differ among genotypes in other plant species (Navas-Lopez et al. 2019; Danti et al. 2022; Christiaens et al. 2014). Differences between genotypes in growth and reproduction may be partially explained by differences in photosynthate availability, as was shown, for example, in *Amaranthus hybridus* (Arntz et al. 2002). Genotype effects are mostly studied in crop plant species. Our findings show that the maternal genotype also plays an important role in non-crop species. Moreover, recently it has been demonstrated that the chemodiversity of specialized compounds other than terpenoids partially depends on the maternal genotype of *T. vulgare* (Dussarrat et al. 2023). Thus, the maternal genotype should be accounted for in experiments studying chemodiversity.

We had also predicted that plants growing in heterogenous plots may be chemically less diverse compared to plants growing in homogenous plots. In line with our prediction, at least plants of the Aacet chemotype displayed such a pattern with regard to the FHD_ind_ of terpenoids. The driving factor behind this result might again be differences in herbivore damage. Herbivores can induce qualitative and quantitative changes in the composition of specialized metabolites in various plant species (Bustos-Segura and Foley 2018; McCormick et al. 2019; Paré and Tumlinson 1997). Plants of the Aacet chemotype may show a lower chemodiversity because of a lower induction of terpenoids due to reduced herbivory, mediated by associational resistance in heterogenous neighborhoods.

Our experimental design allowed to test for phenotypic plasticity, since we used two clones per plant individual, one growing in the homogenous and the other growing in the adjacent heterogenous plot. The results highlight striking differences for reproduction-related plant traits caused by the specific environment a clone is experiencing. Moreover, supporting our hypothesis that plants with higher chemodiversity show an enhanced growth, we found a slight trend toward a positive correlation between FHD_ind_ and numbers of shoots and leaves in one or both years (not significant after correction for multiple testing). This finding is particularly interesting as it indicates that intraspecific chemodiversity may have a similar association to aboveground biomass as plant species biodiversity. In biodiverse neighborhoods, plants produce more aboveground biomass compared to those in monoculture neighborhoods (Prieto et al. 2015).

After correction for multiple testing, the FHD_ind_ and its individual components showed no significant correlation with any growth- and reproduction-related plant traits. This may indicate that chemodiversity is a dimension of the multivariate phenotype of a plant that is independent from growth and reproduction. However, correlations that were significant before *p* value adjustment may indicate potential relationships. For example, depending on the time-point of the year, different distinct components of FHD and leaf number showed a slight potential relationship, which may hint to different herbivore abundance throughout these time-points. Indeed, in June 2021, aphid abundances peaked (Ziaja and Müller 2023), while in August 2020, aphid colonies on the same *T. vulgare* experimental field had declined already (unpublished data), impacting potentially investment into different resources. Plants with a higher disparity in their leaf terpenoid profile also showed by trend a higher maximum number of flower heads in 2020 and 2021. An increase in disparity is considered to increase the levels of synergistic combinations (Liu and Zhao 2016) or increase the number of interactions with other organisms (Petrén et al. 2023, 2024). FHD_ind_ showed by tendency a positive correlation with the N-content and a negative with the C-to-N ratio, the latter also found for compound richness. Thus, our results indicate that terpenoid chemodiversity may be limited rather by the N than the C content. More research on how intraspecific chemodiversity and its individual components may be linked to the growth and reproduction of plants and whether allocation costs may become apparent under resource-limited conditions is needed.

Finally, we found significant correlations between the leaf terpenoid and metabolite richness. These findings indicate that chemodiversity in *T. vulgare* is to a certain degree integrated across different matrices and compound groups. Some of the terpenoids were likely also measured by LC–MS and thus contributed to the overall metabolite richness, which was much higher than the terpenoid richness alone. In another study of LC–MS fingerprints of different *T. vulgare* chemotypes, no association between terpene chemotypes and metabolite fingerprints, or metabotypes, was found (Clancy et al. 2018). This may be explained by the fact that the maternal source of the origin, or genotype, is a more pronounced source of chemical variation than the chemotype (Dussarrat et al. 2023).

In conclusion, an enhanced chemodiversity revealed similar positive relationships with plant performance traits as have been found for biodiversity. An enhanced chemodiversity may therefore likewise result in higher growth and resilience of crop plants. More research is needed to disentangle the roles of intraspecific chemodiversity and genetic diversity in shaping a plant’s phenotype and ultimately their contribution to plant fitness.

## Supplementary Information

Below is the link to the electronic supplementary material.

Supplementary file1 (XLSX 363 KB) Supplement Table S1: Physical parameters and nutrient availability across the experimental common garden site.

Supplementary file2 (XLSX 13 KB) Supplement Table S2: Results of paired statistical tests for each recorded plant trait tested for chemotypes between heterogenous and homogenous plots.

## Data Availability

The raw and processed field data (aphid scores, morphological characterization, and terpenoid analysis) are stored together with the R scripts in the github repository https://gitlab.ub.uni-bielefeld.de/dozi/commongarden-performance-2020-2021.

## Code availability

A virtual environment of the Rstudio project including the packages and their respective version numbers is available on github (https://gitlab.ub.uni-bielefeld.de/dozi/commongarden-performance-2020-2021).
